# Global PARITY: Study Design for a Multi-Centered, International Point Prevalence Study to Estimate the Burden of Pediatric Acute Critical Illness in Resource-Limited Settings

**DOI:** 10.3389/fped.2021.793326

**Published:** 2022-01-28

**Authors:** Qalab Abbas, Adrian Holloway, Paula Caporal, Eliana López-Barón, Asya Agulnik, Kenneth E. Remy, John A. Appiah, Jonah Attebery, Ericka L. Fink, Jan Hau Lee, Shubhada Hooli, Niranjan Kissoon, Erika Miller, Srinivas Murthy, Fiona Muttalib, Katie Nielsen, Maria Puerto-Torres, Karla Rodrigues, Firas Sakaan, Adriana Teixeira Rodrigues, Erica A. Tabor, Amelie von Saint Andre-von Arnim, Matthew O. Wiens, William Blackwelder, David He, Teresa B. Kortz, Adnan T. Bhutta

**Affiliations:** ^1^Department of Pediatrics and Child Health, Aga Khan University Karachi, Karachi, Pakistan; ^2^Division of Pediatric Critical Care Medicine, Department of Pediatrics, University of Maryland Baltimore, Baltimore, MD, United States; ^3^Hospital Interzonal Especializado en Pediatría “Sor María Ludovica”, La Plata, Argentina; ^4^Red Colaborativa Pediátrica de Latinoamérica (LARed Network), Buenos Aires, Argentina; ^5^Hospital Pablo Tobón Uribe, Unidad de Cuidado Crítico Pediátrico, Medellín, Colombia; ^6^Department of Global Pediatric Medicine, St. Jude Children's Research Hospital, Memphis, TN, United States; ^7^Division of Critical Care Medicine, Department of Pediatrics, Washington University School of Medicine, St. Louis, MO, United States; ^8^Division of Pulmonary and Critical Care Medicine, Department of Internal Medicine, University Hospitals of Cleveland and Rainbow Babies and Children's Hospital, Case Western Reserve University School of Medicine, Cleveland, OH, United States; ^9^Pediatric Intensive Care Unit, Komfo Anokye Teaching Hospital, Kumasi, Ghana; ^10^Department of Pediatrics, Section of Pediatric Critical Care, University of Colorado, Aurora, CO, United States; ^11^Division of Pediatric Critical Care Medicine, Department of Critical Care Medicine, UPMC Children's Hospital of Pittsburgh, Pittsburgh, PA, United States; ^12^Children's Intensive Care Unit, KK Women's and Children's Hospital, Singapore, Singapore; ^13^SingHealth Duke-NUS Global Health Institute, Singapore, Singapore; ^14^Division of Pediatric Critical Care, Department of Pediatrics, BC Children's Hospital, The University of British Columbia, Vancouver, BC, Canada; ^15^Department of Pediatrics, Section of Pediatric Emergency Medicine, Baylor College of Medicine, Texas Children's Hospital, Houston, TX, United States; ^16^Division of Pediatric Critical Care, Department of Pediatrics, University of Washington, Seattle Children's, Seattle, WA, United States; ^17^Department of Pediatrics, Hospital das Clínicas da UFMG/EBSERH, Universidade Federal de Minas Gerais, Belo Horizonte, Brazil; ^18^Pennsylvania State University, State College, PA, United States; ^19^Center for Child Health at BC Children's Hospital, The University of British Columbia, Vancouver, BC, Canada; ^20^Department of Pediatrics, Mbarara University of Science and Technology, Mbarara, Uganda; ^21^Department of Anesthesiology, Pharmacology and Therapeutics, Faculty of Medicine, The University of British Columbia, Vancouver, BC, Canada; ^22^Department of Epidemiology and Public Health, University of Maryland, Baltimore, MD, United States; ^23^Analytical Solutions Group, Inc., North Potomac, MD, United States; ^24^Division of Critical Care, Department of Pediatrics, University of California, San Francisco, San Francisco, CA, United States; ^25^Institute for Global Health Sciences, University of California, San Francisco, San Francisco, CA, United States; ^26^Center for Vaccine Development and Global Health, University of Maryland, Baltimore, MD, United States

**Keywords:** pediatric critical illness, acute pediatric care, critical care, outcome, low-and lower-middle-income countries, resource utilization, low resource setting

## Abstract

**Background:**

The burden of pediatric critical illness and resource utilization by children with critical illness in resource limited settings (RLS) are largely unknown. Without specific data that captures key aspects of critical illness, disease presentation, and resource utilization for pediatric populations in RLS, development of a contextual framework for appropriate, evidence-based interventions to guide allocation of limited but available resources is challenging. We present this methods paper which describes our efforts to determine the prevalence, etiology, hospital outcomes, and resource utilization associated with pediatric acute, critical illness in RLS globally.

**Methods:**

We will conduct a prospective, observational, multicenter, multinational point prevalence study in sixty-one participating RLS hospitals from North, Central and South America, Africa, Middle East and South Asia with four sampling time points over a 12-month period. Children aged 29 days to 14 years evaluated for acute illness or injury in an emergency department) or directly admitted to an inpatient unit will be enrolled and followed for hospital outcomes and resource utilization for the first seven days of hospitalization. The primary outcome will be prevalence of acute critical illness, which Global PARITY has defined as death within 48 hours of presentation to the hospital, including ED mortality; or admission/transfer to an HDU or ICU; or transfer to another institution for a higher level-of-care; or receiving critical care-level interventions (vasopressor infusion, invasive mechanical ventilation, non-invasive mechanical ventilation) regardless of location in the hospital, among children presenting to the hospital. Secondary outcomes include etiology of critical illness, in-hospital mortality, cause of death, resource utilization, length of hospital stay, and change in neurocognitive status. Data will be managed via REDCap, aggregated, and analyzed across sites.

**Discussion:**

This study is expected to address the current gap in understanding of the burden, etiology, resource utilization and outcomes associated with pediatric acute and critical illness in RLS. These data are crucial to inform future research and clinical management decisions and to improve global pediatric hospital outcomes.

## Introduction

Over 80 percent of the annual 6.4 million global deaths in children less than 14 years of age occur in low- and middle-income countries (LMICs) with limited resources (1). These deaths are predominantly a result of acute illnesses – sepsis, pneumonia, and trauma – that can be successfully managed with basic, intensive care interventions, such as fluid resuscitation, ventilator support, and transfusion of blood products. Unfortunately, though pediatric acute critical illnesses are the leading causes of death and disability for children outside of the neonatal period globally, acute and critical care services are not universally available in resource-limited settings (RLS) (2–7). A lack of acute and critical care resources is directly associated with worse outcomes, including increased mortality, in children (6–12). Furthermore, this global disparity not only exists with respect to available resources, but also in the availability of data. The true burden and incidence of pediatric acute critical illness is unknown (2, 3, 7, 13–17). Without specific data that captures etiology of acute critical illness and resource utilization in RLS, we cannot develop a contextual framework for appropriate, evidence-based interventions, or appropriately allocate limited but available resources in RLS.

Point prevalence studies are a valuable study design to prospectively gather individual-level data, determine disease prevalence, and measure variability in outcomes and resource utilization across many geographic regions and healthcare settings. Recently, there have been several global point prevalence studies to determine the prevalence of specific pediatric acute critical illnesses: the Pediatric Acute Lung Injury Ventilation (PALIVE) and Pediatric Acute Respiratory Distress Syndrome Incidence and Epidemiology (PARDIE) studies estimated the prevalence of acute pediatric lung injury (10, 15) the International Survey of Critically Ill Children with Acute Neurological Insults (PANGEA) estimated the prevalence of new neurologic injury due to a variety of etiologies (6); and the Sepsis Prevalence, Outcomes, and Therapies (SPROUT) study estimated the prevalence of severe pediatric sepsis (18). While each of these studies contributed significant knowledge about specific critical illnesses, they failed to capture the true global burden of disease and resource utilization within and across LMICs. These studies also required Pediatric Intensive Care Unit (PICU) admission as an inclusion criterion, limiting participation to hospitals with intensive care units. This likely resulted in an underestimation of disease prevalence and mortality in RLS where critical illness is often managed outside of formal intensive care units (2). Additionally, there is significant overlap between illnesses (e.g., pneumonia is a frequent cause of sepsis) and resources required (e.g., mechanical ventilation may be required to support trauma and septic patients) to treat pediatric acute critical illness; therefore, a narrow, disease-specific focus fails to capture both the burden of acute critical illness overall nor does it provide a realistic estimate of resource required to deliver critical care to these patients.

For these reasons the true burden of and outcomes associated with pediatric acute critical illness in RLS have not been previously characterized. As a result, the overall health impact of pediatric acute critical illness in RLS is not known. To better understand the burden of pediatric acute critical illness and associated resource utilization in RLS, we propose the **P**ediatric **A**cute c**R**itical **I**llness point prevalence s**T**ud**Y**; Global PARITY. The overarching objectives of the study are to 1) engage the global pediatric critical care community to establish baseline frequencies and outcomes of common conditions leading to morbidity and mortality of children in RLS, 2) inform a prospective research agenda to challenge the status quo and discover breakthroughs in care to improve pediatric outcomes globally, and 3) measure the burden of pediatric acute critical illness in RLS. Addressing these data gaps are a crucial first to set future clinical research, health delivery, and resource allocation priorities for RLS globally.

## Methods

### Study Design and Setting

A prospective, observational, multicenter, multinational point prevalence study will be conducted in resource-limited hospitals in North, Central, and South America, Africa, the Middle East, and South Asia over four sampling time periods to capture seasonal variation. The specific aims of this study are to determine 1) the etiology and prevalence of pediatric acute critical illness among children presenting to participating hospitals in RLS; 2) measure hospital outcomes (mortality, length of stay) in children with acute critical illness in RLS; 3) determine hospital resource utilization by children with acute critical illness; and 4) determine the current resources available to provide acute critical care across RLS.

**Figure 1:**
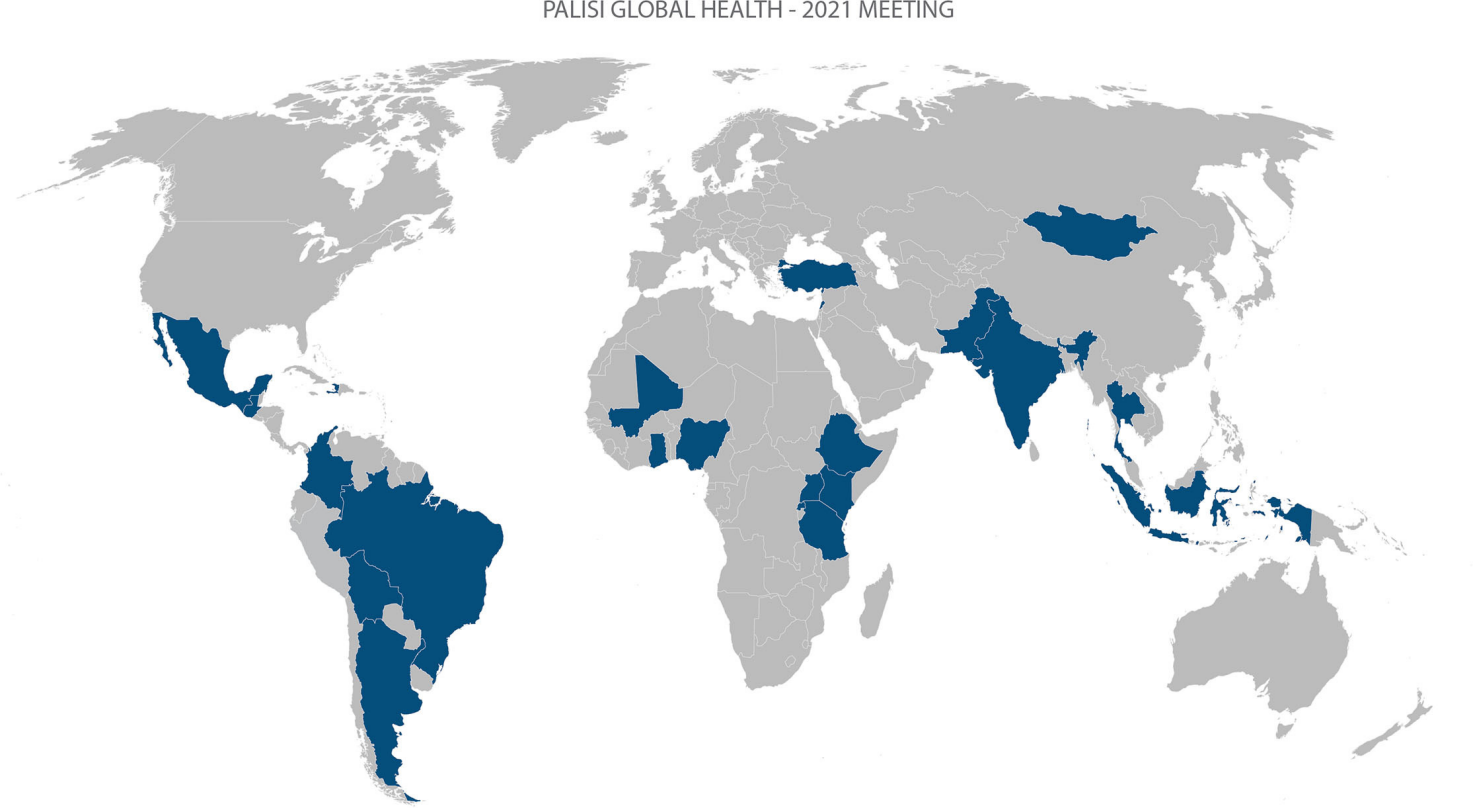
World map of participating sites.

RLS are characterized by a lack of funds to cover health care costs, resulting in: limited access to medication, equipment, supplies, and devices; less-developed infrastructure; and fewer or less-trained personnel. Resource limitations at each site will be assessed by a separate Hospital Resource Survey (7). Eligible sites will: self-identify as a RLS; be an acute care hospital; provide emergency and inpatient care to a general population of children with acute illnesses (i.e., not a specialty hospital); have a reliable internet connection or cellular service for uploading data; have a member of the local research team who can communicate in and understand English; have an established Institutional Review Board (IRB) or ethical approval process; have previous experience with clinical research and data collection; have the ability to support or apply for support for study-related costs. Hospitals will be recruited via established relationships from the Global Health subgroup of the Pediatric Acute Lung Injury and Sepsis Investigators' (PALISI) Research Network (www.palisiglobalhealth.org), the St. Jude Global Critical Care Program (www.stjude.org/global), and the Red Colaborativa Pediátrica de Latinoamérica (LARed Network). At time of this manuscript, 61 sites from 27countries in 8 regions have committed to participation (Table 1, Figure 1). The study is coordinated by the Department of Pediatrics at the University of Maryland and has been deemed exempt by the University of Maryland Institutional Review Board (IRB, HP-00086107).

**Table 1:** Participating sites by country and global PARITY-designated region.

**Country**	**Number of participating sites**	**Global PARITY region**
Argentina	5	Spanish-speaking South America
Barbados	1	North America/Caribbean/Central America
Bolivia	1	Spanish-speaking South America
Brazil	1	Portuguese-speaking South America
Colombia	12	Spanish-speaking South America
Ethiopia	2	West Africa/Ethiopia
Ghana	8	West Africa/Ethiopia
Guatemala	1	North America/Caribbean/Central America
Haiti	1	Francophone Countries
India	2	Middle East/India/Pakistan
Indonesia	1	Southeast Asia
Kenya	1	East/Central Africa
Lebanon	1	Middle East/India/Pakistan
Malaysia	1	Southeast Asia
Mali	1	Francophone Countries
Mexico	2	North America/Caribbean/Central America
Mongolia	1	Southeast Asia
Nigeria	3	West Africa/Ethiopia
Pakistan	3	Middle East/India/Pakistan
Paraguay	1	Spanish-speaking South America
Peru	2	Spanish-speaking South America
Rwanda	3	East/Central Africa
Tanzania	1	East/Central Africa
Thailand	1	Southeast Asia
Turkey	1	Middle East/India/Pakistan
Uganda	2	East/Central Africa
Uruguay	2	Spanish-speaking South America

All children presenting to the Emergency Department (ED), or equivalent acute hospital receiving unit, or who are directly admitted to a participating site hospital will be screened during a 24-h period on four separate days within four epochs over a 12-month period for inclusion and exclusion criteria. Inclusion criteria will include children aged 29 days to 14 years of age evaluated in the ED for an acute illness or injury or directly admitted to an inpatient unit. Children who are evaluated in the ED and discharged home after evaluation, who die in the ED, or are evaluated and transferred to a higher level of care will be included. This number will serve as the total population (denominator) to calculate the prevalence.

Children presenting for a scheduled follow-up visit, vaccinations, suture removal (or other non-acute complaint), children with a corrected gestational age less than 42 weeks, and children who present to the ED and are pronounced dead on arrival will be excluded. Neonates and infants up to 28 days will not be included in this study as the etiology of critical illness and resource requirements to manage neonates and young infants differs significantly from that of older children. Since the upper age limit of what defines a pediatric patient varies by site and region, 14 years was chosen as the upper limit of age, as patients 14 years and younger are generally considered to be children regardless of setting. All children meeting inclusion criteria and no exclusion criteria will be enrolled. Due to the IRB exempt status, consent is not required.

Patients who are admitted to the hospital (e.g., general pediatric ward, high dependency unit [HDU], or intensive care unit [ICU]) either directly or through the ED will be followed daily for up to 7 days to determine daily resource utilization. Admitted patients will also be followed until the time of discharge, death, transfer or hospital day 30, whichever occurs first, to determine hospital outcomes. These admitted patients will be the numerator to calculate the overall prevalence of hospitalizations.

### Data Collection and Management

All data will be collected and managed via REDCap (Research Electronic Data Capture); a secure, web-based application and electronic data capture tool hosted at the University of Maryland (19, 20). No patient-identifying data will be collected. Prior to data collection, a pilot will be conducted at each site to identify challenges in data acquisition and to test study procedures. Study data will be collected using a hospital resource survey, an initial intake survey, a daily resource utilization survey (to be completed daily from the day of presentation up to hospital day seven), and a final outcomes survey (see Supplementary Material). The hospital resource survey is an adapted version of a previously published (7) survey that aims to assess aspects of resource availability, the presence of a basic research infrastructure including ethical and/or IRB approval mechanisms, and the availability of a local site principal investigator (PI). The following data will be collected from the medical record, per exempt status of the IRB, during the study: hospital characteristics including average number of patient encounters and admissions, types of inpatient units, available human resources, available infrastructure including healthcare devices, medications and laboratory resources; patient characteristics including severity of illness, anthropometrics (weight, height, mid-upper arm circumference), comorbidities (HIV status, congenital heart disease, malnutrition), presenting vital signs, routine laboratory test results, imaging results, and the Pediatric Overall Performance Category (POPC) (5) prior to the current illness; hospital resource utilization including use of blood transfusion, fluid bolus, vasoactive agents, non-invasive positive pressure ventilation, oxygen, mechanical ventilation, ICU admission, and antibiotic administration; and outcomes including discharge home, transfer to a higher level of care within the hospital, transfer to another hospital, death, final hospital diagnosis, length of stay, cause of death (if applicable), and the POPC at the time of discharge. For the selected case report forms, see supplements entitled 1) Initial Intake Survey, 2) Daily Assessment Survey, 3) Final Outcomes Survey.

### Outcomes

The primary outcome of interest is prevalence of pediatric acute critical illness, defined as death within 48 h of presentation to the hospital, including ED mortality; or admission/transfer to an HDU or ICU; or transfer to another institution for a higher level-of-care; or receiving critical care-level interventions (vasopressor infusion, invasive mechanical ventilation, non-invasive mechanical ventilation) regardless of location in the hospital. Secondary outcomes include etiology of critical illness, in-hospital mortality, cause of death, resource utilization, length of hospital stay, and change in neurocognitive status from premorbid state from admission to discharge POPC.

### Statistical Analysis

Data will be analyzed on all subjects who meet inclusion criteria. Descriptive analyses will summarize population-level information. Regression modeling will be used to explore risk factors associated with critical illness (primary outcome), in-hospital mortality, resource utilization, length of hospital stay, and change in neurocognitive status from baseline (secondary outcomes). These analyses will include adjustments for clustering (facility-level, region-level), time-varying variables, and time-invariant variables. Variables will be chosen for evaluation in multivariable models based on their empirical significance in the literature (age, sex, severity of illness, HIV status, anemia, malnutrition) and their performance in univariable models.

### Secondary Analyses

Owing to the international and collaborative nature inherent to this study, investigators participating in this study were able to submit proposals for secondary analysis. Proposals for secondary analysis were requested from participating site investigators to query the data generated by Global PARITY to address important questions and gaps not addressed by primary and secondary outcomes of the study. The proposals were reviewed by the Scientific Committee of the Global Health Subgroup; and, a total of 15 proposals were accepted. The list of approved secondary analyses are listed in Table 2.

**Table 2:** Global parity secondary analyses.

**Lead investigators**	**Location**	**Topic or Theme**
1. Asya Agulnik	Global	Burden of critical illness in cancer compared to other patients
2. Enkhtur Sh, Solongo.O, Dulamragchaa.Ch	Mongolia	Epidemiology and outcomes for pediatric acute respiratory distress, sepsis and sepsis-like diseases
3. Shubhada Hooli, Christian Umuhoza	Global	Prediction modeling and scoring systems for mortality in critical illlness
4. Kandamaran Krishnamurthy, Seetharaman Hariharan	Caribbean	Ways to improve education with ethical dilemmas especially when futility reached
5. Onah Stanley et al.	Nigeria	Metabolic derangements and pathogen specific diseases
6.Sofia Esposto et al.	Global	Time to antibiotics in pneumonia
7. Fiona Muttalib	Global	Association between resource availability at the sites and resource utilization/outcomes
8. Teresa Kortz	Global	Predictive success of existing clinical scores developed for RMICs at identifying children at risk of death
9. John Appiah, Adrian Holloway	Global	Blood transfusion delivery in sepsis and influence on patient outcomes
10. Ariet Figueroa et al.	Global	Early measurement of Age adjusted Shock Index to predict outcomes in non-trauma pediatric patients
11. Madiha Raees, Ericka Fink	Global	Epidemiology, resource use, and outcomes of children with neurocritical illness
12. Sofia Esposto et al.	Global	Characteristics of patients and the factors that determine the onset of empiric antibiotic therapy in sepsis
13. Sebastián González-Dambrauskas et al.	Latin America Caribbean	Outcomes and resources between two geographical different locations
14. Tigist Bacha Heye	Global	Presentation, Epidemiology, and Outcomes in Sepsis
15. Kenneth Remy, Tex Kissoon	Global	PP of sepsis, the presence of adequate antimicrobials, factors leading toward increased sepsis burden, and understand comparative mortality associated with certain available supportive therapies in each LMIC for patients with sepsis.

## Discussion

Global PARITY will address the current gap in knowledge regarding the burden of pediatric acute critical illness and hospital resource utilization in RLS. In contrast to previous point prevalence studies estimating the prevalence of specific pediatric critical illnesses (e.g., ARDS, sepsis), Global PARITY aims to measure the burden of all pediatric acute critical illness. Additionally, by expanding the definition of critical illness to include PICU admission, intensive care-level resource utilization, and/or early hospital mortality, the Global PARITY definition is more inclusive and likely to capture critical illness managed in hospitals without formal intensive care units. To our knowledge, this is the first global pediatric point prevalence study to include settings without formal intensive care services and aimed at measuring the prevalence of pediatric critical illness as one entity instead of separate, individual diagnoses.

There are some anticipated limitations to our study. Our study shares limitations common to all point prevalence studies, including inability to account for prehospital mortality or resource utilization. This may result in an underestimation of disease prevalence and resource requirements, especially in those that have a fulminant course or in patients who lack quick access to a hospital setting. Likewise, while the Global PARITY definition of acute critical illness is more inclusive than prior global pediatric point prevalence studies, it has not been previously studied or validated. It is possible that this definition may over or underestimate the true burden of critical illness. However, similar definitions have been used in other multicenter studies (19, 21) in RLS, and we expect to capture the majority of critical illness and associated hospital resource utilization in these patients. Further, while we tried our best to recruit centers from a wide range of countries and regions, we know that there can be disparities within countries based on region, socioeconomic status and type of healthcare facility (such as public vs. private). Thus, our study is still susceptible to selection bias and participating centers may not fully represent the epidemiology of pediatric acute critical illness or resource utilization patterns in a given country or region. Additionally, while we broadened eligible site criteria to include hospitals with or without a formal intensive care unit, the current site requirements (English language, resources, internet connectivity, available Principal Investigator) may still be too burdensome for some sites in RLS to participate. As a result, our study may underestimate the true impact of pediatric acute critical illness, as non-participating sites are likely more resource-limited than those able to meet study criteria. To address this limitation, we are applying for pilot funds to help defer study-related costs at participating sites. We also plan to further explore the reasons site non-participation in future studies. Regardless of these limitations, the results of our study will be the starting point for a generation of urgently need new research and interventional studies.

Global PARITY represents a unique opportunity to engage pediatric clinicians across the world. Only with a large, concerted effort can we, as a global pediatric community, start to understand the spectrum of acute critical illness and its association with childhood morbidity and mortality across resource-variable settings. The results of Global PARITY will inform a prospective, inclusive, global research agenda that includes children around the world, regardless of local resource availability. The data from this study will challenge the status quo and move us toward achieving the long-term goal to develop a body of evidence to support basic, universal, cost-effective critical care interventions appropriate for all settings, especially those with resource limitations. The implementation of such interventions could then be used for targeted capacity-building across resource-limited settings and has the potential to significantly reduce childhood morbidity and mortality due to acute critical illness globally.

## Data Availability Statement

The original contributions presented in the study are included in the article/Supplementary Material, further inquiries can be directed to the corresponding author/s.

## Author Contributions

All authors participated in the development, writing, and editing of the manuscript.

## Funding

Research effort to create this publication was supported by the National Institute of Allergy and Infectious Diseases of the National Institutes of Health under award number K23AI144029 (TK).

## Conflict of Interest

DH is the owner of Analytical Solutions Group, Inc. The remaining authors declare that the research was conducted in the absence of any commercial or financial relationships that could be construed as a potential conflict of interest.

## Publisher's Note

All claims expressed in this article are solely those of the authors and do not necessarily represent those of their affiliated organizations, or those of the publisher, the editors and the reviewers. Any product that may be evaluated in this article, or claim that may be made by its manufacturer, is not guaranteed or endorsed by the publisher.
