# Supplementary material for: Global PARITY: Study Design for a Multi-Centered, International Point Prevalence Study to Estimate the Burden of Pediatric Acute Critical Illness in Resource-Limited Settings
Source: Front Pediatr. 2022 Jan 28;9:793326. doi: 10.3389/fped.2021.793326 (PMC8835113; doi:10.3389/fped.2021.793326)
Supplement: Supplementary file 1 [file Data_Sheet_1.PDF]

Daily Assessment Survey

record ID field
leave blank ______________________

Study tracking

Your Initials ______________________

Enter Patient ID ______________________

This is the REGIONAL code (XX), followed by your SITE NUMBER (12), followed by the PATIENT NUMBER (000)

XX-12-000

Hospital Day
- ◯ 0 (day of presentation/admission)
- ◯ 1
- ◯ 2
- ◯ 3
- ◯ 4
- ◯ 5
- ◯ 6
- ◯ 7

Patient Location at the beginning and end of the day

	Emergency Department	Inpatient Ward	High-Dependency Unit (HDU)	Intermediate Care Unit (IMCU)	Intensive Care Unit (ICU)	Operating room (OR)	Morgue (dead)	Discharged/ transferred/ AMA	Other
Patient location at the START (0800) of the DAY	◯	◯	◯	◯	◯	◯	◯	◯	◯
Patient location at the END (2000) of the DAY	◯	◯	◯	◯	◯	◯	◯	◯	◯

Laboratory and Diagnostic Data

Select "yes" if a value is recorded in the medical record; "no" if not measured or recorded.

First measurement after presentation to the emergency department, on admission to the hospital if direct admit, or first value of the day if the patient is admitted. All CBC (Hgb, platelets) and chemistries (Cr, BUN) measurements should be from the same lab draw.

	No	Yes
White blood cell count (WBC)	◯	◯

Absolute lymphocyte percent (ALC)	◯	◯
Hemoglobin (HGB)	◯	◯
Platelet count	◯	◯
Glucose	◯	◯
Blood urea nitrogen (BUN)	◯	◯
AST/SGOT	◯	◯
ALT/SGPT	◯	◯
Bilirubin	◯	◯
Lactate dehydrogenase (LDH)	◯	◯
Creatinine	◯	◯
Serum pH	◯	◯
Lactate	◯	◯
Partial thromboplastin time (PTT)	◯	◯
INR	◯	◯
D-dimer	◯	◯
Fibrinogen	◯	◯
Ferritin	◯	◯
Erythrocyte sedimentation rate (ESR)	◯	◯
C-reactive protein (CRP)	◯	◯
Brain natriuretic peptide (BNP)	◯	◯
Head imaging	◯	◯
Chest imaging	◯	◯
Abdominal imaging	◯	◯
Echocardiogram	◯	◯

Total white blood cell count (WBC)

____
(in 10^9)

Absolute LYMPHOCYTE count (%)

____

Serum hemoglobin

____

Hemoglobin units

◯ g/DL
◯ mmol/L

Platelet Count

____
(in 10^9)

Glucose

____

Add "0" for undetectably LOW
Add "800" of undetectably HIGH

Glucose units	◯ mg/dL<br>◯ mmol/L
Blood Urea Nitrogen	__________________
Blood urea nitrogen units	◯ mg/dL<br>◯ mmol/L
Serum AST/SGOT<br><br>In IU	__________________<br>(in IU/L)
Serum ALT/SGPT<br><br>In IU	__________________<br>(in IU/L)
Serum Bilirubin (Total)	__________________
Bilirubin Units	◯ mg/dL<br>◯ SI
Lactate dehydrogenase (LDH)	__________________
Lactate dehydrogenase units	◯ units/L<br>◯ SI units
Creatinine	__________________
Creatinine units	◯ mg/dL<br>◯ micrmol/L
Serum pH	__________________
pH blood source	◯ Arterial<br>◯ Venous<br>◯ Capillary<br>◯ Unknown
Serum Lactate	__________________
Lactate blood source	◯ Arterial<br>◯ Venous<br>◯ Capillary<br>◯ Unknown
Lactate blood units	◯ mg/dL<br>◯ mmol/L (SI units)

Partial Thromboplastin Time (PTT)	______________________ (in seconds)
INR	______________________
D-dimer	______________________
D-Dimer units	◯ NANOgram/mL<br>◯ MICROgram/mL
Fibrinogen	______________________
Fibrinogen units	◯ mg/dL<br>◯ g/L (SI unit)
Ferritin	______________________
Ferritin Units	◯ MICROgrams/L<br>◯ NANOgrams/ml
Erythrocyte sedimentation rate in mm/hr	______________________
C reactive protein	______________________
C reactive protein units	◯ MILIgrams/L (SI units)<br>◯ MICROgrams/mL
Brain natriuretic peptide (BNP) in NANOgrams/L	______________________
Head Imaging	☐ Computed tomography (CT)<br>☐ Magnetic resonance imaging (MRI)<br>☐ Head ultrasound
Head imaging results	___________________________
Chest Imaging	☐ Computed tomography (CT)<br>☐ Magnetic resonance imaging (MRI)<br>☐ Ultrasound<br>☐ Plain x-ray
Please indicate which quadrants on chest imaging contain opacifications. Choose all that apply.	☐ Right Upper Lobe<br>☐ Right Middle/Lower Lobe<br>☐ Left Upper Lobe<br>☐ Left Lower Lobe<br>☐ None

Chest imaging results ________________

Abdominal Imaging

- ☐ Computed tomography (CT)
- ☐ Magnetic resonance imaging (MRI)
- ☐ Ultrasound
- ☐ Plain x-ray

Abdominal imaging results ________________

Echocardiogram Results ________________

Did the patient receive any of the following therapies/interventions?

Select "yes" for all of the following therapies administered during that hospital day (time of presentation/0000 to 2359)

	No	Yes
Continuous Sedation >4 hrs	○	○
Invasive Mechanical Ventilation	○	○
Non-Invasive Postive Pressure (CPAP, BiPAP, High-flow)	○	○
Simple or Low-Flow Oxygen Therapy	○	○
Fluid Bolus	○	○
Blood or Blood Product Transfusion	○	○
Vasoactives	○	○
Corticosteroids	○	○
Antibiotic therapy	○	○
Anti-malarial therapy	○	○
Anti-viral therapy	○	○
Anti-fungal therapy	○	○
Anticoagulation (low-molecular weight heparin, heparin infusion)	○	○
Dialysis (any type)	○	○
Procedure (bedside or surgical)	○	○
Chest Compressions	○	○

Does this patient have a suspected or proven diagnosis of COVID-10 or MIS-C?

- ○ Yes
- ○ No

If acute COVID-19 or MIS-C associated with COVID-19, which of the following therapies were used?	☐ Corticosteroid<br>☐ Remdesivir<br>☐ Tociluzimab<br>☐ Monoclonal antibodies<br>☐ IVIG<br>☐ Anticoagulation (low-molecular weight heparin, heparin infusion)<br>☐ Aspirin<br>☐ None of the above<br>☐ Other
List other therapy administered for COVID or MIS-C	______________________
Select which corticosteroid (select all the apply)	☐ Hydrocortisone<br>☐ Dexamethasone<br>☐ Methylprednisolone<br>☐ Prednisone<br>☐ Other
List other steroid	______________________
Select the type of anti-coagulation	◯ Prophylactic<br>◯ Therapeutic<br>◯ Not documented
Which medications were used for continuous sedation?	☐ Fentanyl<br>☐ Remifentanyl or sufentanyl<br>☐ Morphine<br>☐ Hydromorphone<br>☐ Midazolam<br>☐ Alprazolam<br>☐ Lorazepam<br>☐ Dexmedetomidine<br>☐ Propofol<br>☐ Ketamine<br>☐ Inhaled volatile anaesthetics<br>☐ Other
List the other medications if other was selected	____________________________
How long did the patient receive continuous sedation?	◯ < 6hrs<br>◯ 6-12hrs<br>◯ 12-18hrs<br>◯ >18hrs<br>◯ Not documented
If the patient received vasoactives, please indicate the type.	☐ Epinephrine (Adrenalin)<br>☐ Norepinephrine (Noradrenalin)<br>☐ Dopamine<br>☐ Dobutamine<br>☐ Vasopressin<br>☐ Phenylephrine<br>☐ Milrinone<br>☐ Other

If patient received other vasoactives, please list

____

What type of dialysis (select all that apply)

- ☐ Intermittent hemodialysis (HD)
- ☐ Continuous renal replacement therapy (CRRT, CVVH, CVVHD)
- ☐ Peritoneal dialysis
- ☐ Not documented

What procedure(s) did the patient receive (select all that apply)?

- ☐ Central line placement including HD catheter
- ☐ PICC line placement
- ☐ Arterial line placement
- ☐ Lumbar puncture
- ☐ Chest tube/pigtail placement/Thoracentesis
- ☐ Paracentesis/Peritoneal drain placement
- ☐ Extraventricular drain (EVD)/Intracranial pressure monitor (bedside)
- ☐ Bronchoscopy
- ☐ Incision and drainage
- ☐ Interventional or diagnostic procedure (cardiac cath, interventional radiology, EGD, colonoscopy)
- ☐ Surgical procedure in the operating room
- ☐ Other

List interventional procedure here:

____

List surgical procedure here:

____

List other procedure here:

____

pARDS Questions

What was the highest level of respiratory support received by the patient?

- ◯ Invasive mechanical ventilation
- ◯ CPAP or Bipap
- ◯ High Flow Oxygen
- ◯ Simple or Low Flow Oxygen
- ◯ None (was in room air)

What is the oxygen saturation 6 hours after starting mechanical ventilation?

____
(as a %)

What is the fraction of inspired oxygen 6 hours after starting mechanical ventilation?

____
(as a %)

What is the mean airway pressure 6 hours after starting mechanical ventilation?

____
(in cmH20)

If the patient received mechanical ventilation, please indicate the type

- ☐ Conventional
- ☐ High-frequency oscillatory ventilation
- ☐ JET
- ☐ Other

If other mode of invasive mechanical ventilation, please describe

____

If the patient received non-invasive positive pressure ventilation, indicate the type.

- ☐ CPAP
- ☐ Bubble CPAP
- ☐ BiPap
- ☐ High-flow nasal cannula
- ☐ Other

If other type of non-invasive positive pressure, please describe

____

What type of interface is used to provide non-invasive positive pressure ventilation?

- ◯ Nasal cannula/prongs
- ◯ Nasal-only pillows
- ◯ Oro-nasal mask (any mask that covers both nose and mouth)
- ◯ Not documented

If the patient received simple or low-flow oxygen, indicate the type

- ☐ Nasal cannula
- ☐ Face mask of any type (simple, non-rebreather, reservoir)
- ☐ Other
- ☐ Not documented

If other type of low-flow oxygen was used, please describe

____

Vital Signs Available/Recorded at (or closest to) 0800

	No	Yes
Heart Rate	◯	◯
Respiratory Rate	◯	◯
Blood Pressure	◯	◯
Oxygen Saturation	◯	◯
Temperature	◯	◯
AVPU Score (measured or calculable)	◯	◯
Glasgow Coma Scale	◯	◯
Blantyre Coma Scare	◯	◯

Heart Rate

____

Respiratory Rate
in breaths/minute

____

Systolic Blood Pressure
in mmHg

____

Diastolic Blood Pressure
in mmHg

____

Oxygen Saturation	______________________________
Was this saturation obtained while the patient was receiving any source of oxygen?	◯ Yes<br>◯ No<br>◯ Not documented
Temperature<br>in degrees C	______________________________
AVPU Score	◯ Alert<br>◯ Voice<br>◯ Pain<br>◯ Unresponsive
Glasgow Coma Scale: Total Score	______________________________
Glasgow Coma Scale: EYE	◯ 1: Does not open eyes<br>◯ 2: Open eyes in response to pain<br>◯ 3: Open eyes in response to voice<br>◯ 4: Open eyes spontaneously
Glasgow Coma Scale: VERBAL	◯ 1: Makes no sound<br>◯ 2: Makes sound<br>◯ 3: Words<br>◯ 4: Confused, disoriented speech<br>◯ 5: Oriented speech
Glasgow Coma Scale: MOTOR	◯ 1: Makes no movement<br>◯ 2: Abnormal extension to painful stimuli<br>◯ 3: Abnormal flexion to painful stimuli<br>◯ 4: Withdrawal from painful stimuli<br>◯ 5: Localizes to painful stimuli<br>◯ 6: Obeys Commands
Blantyre Coma Scale: Total Score	______________________________
Was the mental status score (AVPU, GCS, BCS) calculated while the patient was on continuous sedation for more than 4 hours?	◯ Yes<br>◯ No<br>◯ Not documented
Number of Stools<br>(if counted)	______________________________
Urine output in ml/kg<br>(if calculated)	______________________________
Fluid balance for the 24 hour period (or since admission if Day 0)<br>Use [-] for negative fluid balance<br>Use [+] for positive fluid balance<br>Or enter Not Recorded	______________________________

Any additional comments
