# Supplementary material for: Global PARITY: Study Design for a Multi-Centered, International Point Prevalence Study to Estimate the Burden of Pediatric Acute Critical Illness in Resource-Limited Settings
Source: Front Pediatr. 2022 Jan 28;9:793326. doi: 10.3389/fped.2021.793326 (PMC8835113; doi:10.3389/fped.2021.793326)
Supplement: Supplementary file 2 [file Data_Sheet_2.PDF]

Final Outcomes Survey

record ID field
leave blank ________________________

Study Tracking

Your Initials ________________________

Enter Patient ID ________________________

This is the REGIONAL code (XX), followed by your SITE NUMBER (12), followed by the PATIENT NUMBER (000)

XX-12-000

Outcome

Hospital Outcome
- ◯ Discharged home
- ◯ Transferred to other facility
- ◯ Death
- ◯ Absconded or left against medical advice
- ◯ Alive and still admitted on Day 31

If transferred, was the patient transferred for a higher level of care?
- ◯ Yes
- ◯ No
- ◯ Not documented

On which Hospital Day did the final outcome occur?
- ◯ 0 (day of presentation/admission)
- ◯ 1
- ◯ 2
- ◯ 3
- ◯ 4
- ◯ 5
- ◯ 6
- ◯ 7
- ◯ Day 8-30
- ◯ > 30 days
- ◯ Not Documented

On what hospital day did the outcome occur? ________________________

Final Diagnoses or Underlying Causes of Death

For patients alive but still admitted on Day 30, select the current working diagnoses

What is the primary discharge diagnosis or underlying cause of death?
- ◯ Communicable and nutritional conditions
- ◯ Non-communicable diseases
- ◯ Injuries
- ◯ Ill-defined or cause unknown

Choose the most appropriate diagnosis

◯ Pneumonia
◯ Bronchiolitis
◯ Upper respiratory tract infection or croup
◯ Tuberculosis
◯ Diarrhea/gastroenteritis
◯ Hepatitis
◯ Measles
◯ Pertussis
◯ Tetanus
◯ Urinary tract infection or pyelonephritis
◯ Acute otitis media
◯ Pharyngitis
◯ HIV/AIDS or AIDS-related illness
◯ Sepsis or septic shock
◯ Acute Malaria
◯ Multisystem Inflammatory Syndrome in Children (MISC)
◯ Acute COVID-19
◯ Any skin or soft tissue infection
◯ Malnutrition
◯ Meningitis or Encephalitis
◯ Fever and neutropenia
◯ Other infectious or parasitic disease

Please indicate specific forms of malnutrition (select all that apply)

☐ Wasting
☐ Failure to thrive
☐ Kwashiorkor
☐ Severe acute malnutrition (SAM)
☐ Stunting
☐ Marasmus
☐ Other
☐ Not documented

List other type of malnutrition

____

Please give details for other infectious cause or parasitic disease

____

Choose the most appropriate diagnosis	◯ Congenital malformations<br>◯ Birth Asphyxia<br>◯ Prematurity<br>◯ Hydrocephalus (with or without a VPS)<br>◯ Stroke<br>◯ Status Epilepticus or seizure<br>◯ Heart Failure<br>◯ Diabetes or related complication (diabetic ketoacidosis, hyperglycemia, hypoglycemia)<br>◯ Bowel obstruction<br>◯ Intussusception<br>◯ Appendicitis<br>◯ Gastrointestinal bleed (upper or lower)<br>◯ Peptic ulcer disease/GERD/Reflux<br>◯ Constipation<br>◯ Pancreatitis<br>◯ Cancer/malignancy<br>◯ Allergies, allergic rhinitis<br>◯ Asthma/Status Asthmaticus<br>◯ Chronic Respiratory or lung Disease<br>◯ Sickle cell disease/anemia or associated complication (acute chest, pain crisis)<br>◯ Hypovolemia/Dehydration<br>◯ Shock<br>◯ Anemia<br>◯ Renal failure or injury<br>◯ Carbon monoxide poisoning<br>◯ Other non-communicable diseases
What is the cancer or oncologic diagnosis?	__________________________________
Indicate whether acute or chronic kidney or renal disease	◯ Acute<br>◯ Chronic<br>◯ Not documented
If shock, please indicate type	◯ Cardiogenic<br>◯ Neurogenic<br>◯ Anaphylactic<br>◯ Hemorrhagic<br>◯ Hypovolemic due to dehydration<br>◯ Obstructive<br>◯ Other<br>◯ Not documented
List other type of shock	____________________________
Indicate the type of stroke	◯ Non-traumatic hemorrhagic stroke<br>◯ Ischemic<br>◯ Other<br>◯ Not documented
List or describe other kind of stroke	__________________________________

If other non-communicable disease, please describe

____

Choose the most appropriate diagnosis

- ◯ Traumatic brain injury
- ◯ Polytrauma
- ◯ Fracture
- ◯ Laceration
- ◯ Non-accidental trauma or child abuse
- ◯ Self-injury or suicide attempt
- ◯ Assault
- ◯ Fall
- ◯ Drowning
- ◯ Poisoning/Ingestion
- ◯ Burn
- ◯ Envonmation by either bite or sting
- ◯ Foreign body aspiration
- ◯ Foreign body ingestion
- ◯ Other injury

If other injury, please describe

____

Please describe ill-defined or undefined cause

____

Would you like to add a SECONDARY discharge diagnosis or underlying cause of death?

- ◯ Yes
- ◯ No

What is the SECONDARY discharge diagnosis or underlying cause of death?

- ◯ Communicable and nutritional conditions
- ◯ Non-communicable diseases
- ◯ Injuries
- ◯ Ill-defined or cause unknown

Choose the most appropriate diagnosis	◯ Pneumonia<br>◯ Bronchiolitis<br>◯ Upper respiratory tract infection or croup<br>◯ Tuberculosis<br>◯ Diarrhea/gastroenteritis<br>◯ Hepatitis<br>◯ Measles<br>◯ Pertussis<br>◯ Tetanus<br>◯ Urinary tract infection or pyelonephritis<br>◯ Acute otitis media<br>◯ Pharyngitis<br>◯ HIV/AIDS or AIDS-related illness<br>◯ Sepsis or septic shock<br>◯ Acute Malaria<br>◯ Multisystem Inflammatory Syndrome in Children (MISC)<br>◯ Acute COVID-19<br>◯ Any skin or soft tissue infection<br>◯ Malnutrition<br>◯ Meningitis or Encephalitis<br>◯ Fever and neutropenia<br>◯ Other infectious or parasitic disease
Please indicate specific forms of malnutrition (select all that apply)	☐ Wasting<br>☐ Failure to thrive<br>☐ Kwashiorkor<br>☐ Severe acute malnutrition (SAM)<br>☐ Stunting<br>☐ Marasmus<br>☐ Other<br>☐ Not documented
List other type of malnutrition	______________________________
Please give details for other infectious cause or parasitic disease	______________________________________

Choose the most appropriate diagnosis

- ◯ Congenital malformations
- ◯ Birth Asphyxia
- ◯ Prematurity
- ◯ Hydrocephalus (with or without a VPS)
- ◯ Stroke
- ◯ Status Epilepticus or seizure
- ◯ Heart Failure
- ◯ Diabetes or related complication (diabetic ketoacidosis, hyperglycemia, hypoglycemia)
- ◯ Bowel obstruction
- ◯ Intussusception
- ◯ Appendicitis
- ◯ Gastrointestinal bleed (upper or lower)
- ◯ Peptic ulcer disease/GERD/Reflux
- ◯ Constipation
- ◯ Pancreatitis
- ◯ Cancer/malignancy
- ◯ Allergies, allergic rhinitis
- ◯ Asthma/Status Asthmaticus
- ◯ Chronic Respiratory or lung Disease
- ◯ Sickle cell disease/anemia or associated complication (acute chest, pain crisis)
- ◯ Hypovolemia/Dehydration
- ◯ Shock
- ◯ Anemia
- ◯ Renal failure or injury
- ◯ Carbon monoxide poisoning
- ◯ Other non-communicable diseases

What is the cancer or oncologic diagnosis?

____

Indicate whether acute or chronic kidney or renal disease

- ◯ Acute
- ◯ Chronic
- ◯ Not documented

If shock, please indicate type

- ◯ Cardiogenic
- ◯ Neurogenic
- ◯ Anaphylactic
- ◯ Hemorrhagic
- ◯ Hypovolemic due to dehydration
- ◯ Obstructive
- ◯ Other
- ◯ Not documented

List other type of shock

____

Indicate the type of stroke

- ◯ Non-traumatic hemorrhagic stroke
- ◯ Ischemic
- ◯ Other
- ◯ Not documented

List or describe other kind of stroke

____

If other non-communicable disease, please describe

____

Choose the most appropriate diagnosis

- ◯ Traumatic brain injury
- ◯ Polytrauma
- ◯ Fracture
- ◯ Laceration
- ◯ Non-accidental trauma or child abuse
- ◯ Self-injury or suicide attempt
- ◯ Assault
- ◯ Fall
- ◯ Drowning
- ◯ Poisoning/Ingestion
- ◯ Burn
- ◯ Envonmation by either bite or sting
- ◯ Foreign body aspiration
- ◯ Foreign body ingestion
- ◯ Other injury

If other injury, please describe

____

Please describe ill-defined or undefined cause

____

Would you like to add a TERTIARY discharge diagnosis or underlying cause of death?

- ◯ Yes
- ◯ No

What is the TERTIARY discharge diagnosis or underlying cause of death?

- ◯ Communicable and nutritional conditions
- ◯ Non-communicable diseases
- ◯ Injuries
- ◯ Ill-defined or cause unknown

Choose the most appropriate diagnosis	◯ Pneumonia<br>◯ Bronchiolitis<br>◯ Upper respiratory tract infection or croup<br>◯ Tuberculosis<br>◯ Diarrhea/gastroenteritis<br>◯ Hepatitis<br>◯ Measles<br>◯ Pertussis<br>◯ Tetanus<br>◯ Urinary tract infection or pyelonephritis<br>◯ Acute otitis media<br>◯ Pharyngitis<br>◯ HIV/AIDS or AIDS-related illness<br>◯ Sepsis or septic shock<br>◯ Acute Malaria<br>◯ Multisystem Inflammatory Syndrome in Children (MISC)<br>◯ Acute COVID-19<br>◯ Any skin or soft tissue infection<br>◯ Malnutrition<br>◯ Meningitis or Encephalitis<br>◯ Fever and neutropenia<br>◯ Other infectious or parasitic disease
Please indicate specific forms of malnutrition (select all that apply)	☐ Wasting<br>☐ Failure to thrive<br>☐ Kwashiorkor<br>☐ Severe acute malnutrition (SAM)<br>☐ Stunting<br>☐ Marasmus<br>☐ Other<br>☐ Not documented
List other type of malnutrition	__________________________________
Please give details for other infectious cause or parasitic disease	____

Choose the most appropriate diagnosis	◯ Congenital malformations<br>◯ Birth Asphyxia<br>◯ Prematurity<br>◯ Hydrocephalus (with or without a VPS)<br>◯ Stroke<br>◯ Status Epilepticus or seizure<br>◯ Heart Failure<br>◯ Diabetes or related complication (diabetic ketoacidosis, hyperglycemia, hypoglycemia)<br>◯ Bowel obstruction<br>◯ Intussusception<br>◯ Appendicitis<br>◯ Gastrointestinal bleed (upper or lower)<br>◯ Peptic ulcer disease/GERD/Reflux<br>◯ Constipation<br>◯ Pancreatitis<br>◯ Cancer/malignancy<br>◯ Allergies, allergic rhinitis<br>◯ Asthma/Status Asthmaticus<br>◯ Chronic Respiratory or lung Disease<br>◯ Sickle cell disease/anemia or associated complication (acute chest, pain crisis)<br>◯ Hypovolemia/Dehydration<br>◯ Shock<br>◯ Anemia<br>◯ Renal failure or injury<br>◯ Carbon monoxide poisoning<br>◯ Other non-communicable diseases
What is the cancer or oncologic diagnosis?	__________________________________
Indicate whether acute or chronic kidney or renal disease	◯ Acute<br>◯ Chronic<br>◯ Not documented
If shock, please indicate type	◯ Cardiogenic<br>◯ Neurogenic<br>◯ Anaphylactic<br>◯ Hemorrhagic<br>◯ Hypovolemic due to dehydration<br>◯ Obstructive<br>◯ Other<br>◯ Not documented
List other type of shock	____________________________
Indicate the type of stroke	◯ Non-traumatic hemorrhagic stroke<br>◯ Ischemic<br>◯ Other<br>◯ Not documented
List or describe other kind of stroke	__________________________________

If other non-communicable disease, please describe

____

Choose the most appropriate diagnosis

◯ Traumatic brain injury
◯ Polytrauma
◯ Fracture
◯ Laceration
◯ Non-accidental trauma or child abuse
◯ Self-injury or suicide attempt
◯ Assault
◯ Fall
◯ Drowning
◯ Poisoning/Ingestion
◯ Burn
◯ Envonmation by either bite or sting
◯ Foreign body aspiration
◯ Foreign body ingestion
◯ Other injury

If other injury, please describe

____

Please describe ill-defined or undefined cause

____

If acute COVID-19 infection of MIS-C associated with COVID-19, how was it diagonsed?

◯ Viral PCR or NAAT (nose swab)
◯ Other rapid test (antigen)
◯ COVID antibody positive
◯ Close association with others with COVID-19

Pathogen identification

Was a definitive pathogen(s) identified during hospitalization?

◯ Yes
◯ No
◯ Not documented

How many pathogens were definitively identified?

◯ 1
◯ 2
◯ 3
◯ 4

Pathogen 1 name:

____

Pathogen 1 source (select all the apply):

☐ Blood
☐ Cerebrospinal fluid (CSF)
☐ Sputum
☐ Urine
☐ Nasopharyx or oropharynx
☐ Abscess
☐ Other

If other source of pathogen, please describe

____

Pathogen 2 name: ______________________________

Pathogen 2 source (select all the apply):
- ☐ Blood
- ☐ Cerebrospinal fluid (CSF)
- ☐ Sputum
- ☐ Urine
- ☐ Nasopharyx or oropharynx
- ☐ Abscess
- ☐ Other

If other source of pathogen, please describe ______________________________________

Pathogen 3 name: ______________________________

Pathogen 3 source (select all the apply):
- ☐ Blood
- ☐ Cerebrospinal fluid (CSF)
- ☐ Sputum
- ☐ Urine
- ☐ Nasopharyx or oropharynx
- ☐ Abscess
- ☐ Other

If other source of pathogen, please describe ______________________________________

Pathogen 4 name: ______________________________

Pathogen 4 source (select all the apply):
- ☐ Blood
- ☐ Cerebrospinal fluid (CSF)
- ☐ Sputum
- ☐ Urine
- ☐ Nasopharyx or oropharynx
- ☐ Abscess
- ☐ Other

If other source of pathogen, please describe ______________________________________

Pediatric Overall Performance Category

Pediatric Overall Performance Category
- ◯ Normal
- ◯ Mild Disability
- ◯ Moderate Disability
- ◯ Severe Disability
- ◯ Coma or vegetative state
- ◯ Brain death
- ◯ Not able to determine

Is disability due to physical or mental injury?	◯ No<br>◯ Disability due to physical injury<br>◯ Disability due to mental injury<br>◯ Disability due to both physical and mental injury<br>◯ Unknown<br>◯ Not documented
Any additional comments	____________________________________
