# Supplementary material for: Global PARITY: Study Design for a Multi-Centered, International Point Prevalence Study to Estimate the Burden of Pediatric Acute Critical Illness in Resource-Limited Settings
Source: Front Pediatr. 2022 Jan 28;9:793326. doi: 10.3389/fped.2021.793326 (PMC8835113; doi:10.3389/fped.2021.793326)
Supplement: Supplementary file 3 [file Data_Sheet_3.PDF]

Initial Intake Survey

record ID field
leave blank ______________________

Study Tracking

Enrollment Day
○ 1
○ 2
○ 3
○ 4

Enter Site ID
(Example: SA26) ______________________

Your Initials ______________________

Enter Patient ID ______________________

This is the REGIONAL code (XX), followed by your SITE NUMBER (12), followed by the PATIENT NUMBER (000)

XX-12-000

Demographic and Anthropometric Data

Biologic Sex
○ Female
○ Male
○ not reported
(what is the patient's assigned sex at birth)

Patient Age
○ Under 2 years
○ Over 2 years

If patient under 2 years of age, write age in months ______________________

If patient over 2 year of age, write age in years ______________________

Weight (kg) ______________________

Height or Length (cm) ______________________
(in cm)

Mid-Upper Arm Circumference (MUAC) (cm) ______________________

Presentation/Admission Data

Was this patient directly admitted or hospitalized bypassing your hospital's emergency department?
- ◯ Yes
- ◯ No

What is the admission source?
- ◯ Operating room
- ◯ Transfer or referral
- ◯ Outpatient source

Admission Location
- ◯ Inpatient Ward
- ◯ High-Dependency Unit (HDU)
- ◯ Intermediate Care Unit (IMCU)
- ◯ Intensive Care Unit (ICU)
- ◯ Other

If other, please describe ______________________

Initial Vital Signs Available/Recorded (Yes or No)

Refers to the first set of available vital signs on presentation/admission
All vital signs/measurements should be from the same assessment

	No	Yes
Heart Rate	◯	◯
Respiratory Rate	◯	◯
Blood Pressure	◯	◯
Oxygen Saturation	◯	◯
Temperature	◯	◯
AVPU (measured or calculable)	◯	◯
Glasgow coma scale (measured or calculable)	◯	◯
Blantyre coma scale (measured or calculable)	◯	◯

Heart Rate ______________________
(in beats per minute)

Respiratory Rate ______________________
(in breaths per minute)

Systolic Blood Pressure ______________________
(in mmHg)

Diastolic Blood Pressure ______________________
(in mmHg)

Oxygen Saturation ______________________
(as a %)

Was this saturation obtained while the patient was receiving any source of oxygen?	◯ Yes<br>◯ No<br>◯ Not documented
Temperature	______________________<br>(degrees Celsius)
AVPU Scale<br>(Alert-Verbal-Pain-Unresponsive)	◯ Alert<br>◯ Verbal<br>◯ Pain<br>◯ Unresponsive
Glasgow Coma Scale: Total score	______________________
Glasgow Coma Scale: EYE	◯ 1: Does not open eyes<br>◯ 2: Open eyes in response to pain<br>◯ 3: Open eyes in response to voice<br>◯ 4: Open eyes spontaneously
Glasgow Coma Scale: VERBAL	◯ 1: Makes no sound<br>◯ 2: Makes sound<br>◯ 3: Words<br>◯ 4: Confused, disoriented speech<br>◯ 5: Oriented speech
Glasgow Coma Scale: MOTOR	◯ 1: Makes no movement<br>◯ 2: Abnormal extension to painful stimuli<br>◯ 3: Abnormal flexion to painful stimuli<br>◯ 4: Withdrawal from painful stimuli<br>◯ 5: Localizes to painful stimuli<br>◯ 6: Obeys Commands
Blantyre Coma Scale: Total score	______________________
Was the mental status score (AVPU, GCS, BCS) calculated while the patient was on continuous sedation for more than 4 hours?	◯ Yes<br>◯ No<br>◯ Not documented

Signs and Symptoms

Select yes if any of the following are listed in the medical record as a symptom or sign in the history of present illness or review of systems at the time of admission

Vomiting Everything	◯ Yes<br>◯ No<br>◯ Not documented
Inability to feed	◯ Yes<br>◯ No<br>◯ Not documented

Seizure or Convulsion (observed or reported)
- ◯ Yes
- ◯ No
- ◯ Not documented

Physical Exam Findings

For all physical exam findings, select yes if the finding is listed in the medical record as a physical finding observed at the time of admission

Sunken Eyes
- ◯ Yes
- ◯ No
- ◯ Not documented

Slow skin pinch
- ◯ Yes
- ◯ No
- ◯ Not documented

Severe Pallor
- ◯ Yes
- ◯ No
- ◯ Not documented

Jaundice
- ◯ Yes
- ◯ No
- ◯ Not documented

Prostration
- ◯ Yes
- ◯ No
- ◯ Not documented

Coma
- ◯ Yes
- ◯ No
- ◯ Not documented

Deep Breathing
- ◯ Yes
- ◯ No
- ◯ Not documented

If there is deep breathing, please describe. Select all that apply
- ☐ Rapid, shallow breathing
- ☐ Nasal flaring
- ☐ Grunting
- ☐ Chest in-drawing
- ☐ Accessory muscle use
- ☐ Obstructed breathing
- ☐ Wheezing
- ☐ Stridor
- ☐ Crepitations
- ☐ Central cyanosis
- ☐ Cough

Capillary Refill Time

____
(in seconds)

Pulse Quality
- ◯ Normal
- ◯ Bounding
- ◯ Thready
- ◯ Not documented

Outcomes and Disposition

Disposition upon Discharge from Emergency Department

◯ Discharged home
◯ Operating Room/Operating Theater
◯ Admitted to Inpatient Service
◯ Transferred to Other Facility
◯ Death
◯ Absconded or left against medical advice

If transferred, was the patient transferred for a higher level of care?

◯ Yes
◯ No
◯ Not documented

Location upon admission to inpatient service

◯ Inpatient Ward
◯ High-Dependency Unit (HDU)
◯ Intermediate Care Unit (IMCU)
◯ Intensive Care Unit (ICU)
◯ Other
◯ Not documented

Please give details if admitted to other inpatient setting

____

Length of Emergency Department Stay

____
(in hours)

Location upon leaving operating room/operating theater

◯ Inpatient Ward
◯ High-Dependency Unit (HDU)
◯ Intermediate Care Unit (IMCU)
◯ Intensive Care Unit (ICU)
◯ Death

Pediatric Overall Performance Categrory

◯ Normal
◯ Mild Disability
◯ Moderate Disability
◯ Severe Disability
◯ Coma or vegetative state
◯ Brain death
◯ Not able to determine

Is disability due to physical or mental injury?

◯ No
◯ Disability due to physical injury
◯ Disability due to mental injury
◯ Disability due to both physical and mental injury
◯ Unknown
◯ Not documented

Final Emergency Department Diagnoses, Admission Diagnoses, or Underlying Causes of Death

Select the most appropriate diagnosis or underlying cause of death from the list that best matches the physician's diagnoses (presumptive, likely, suspected, or final)

What is the primary ED or admission diagnosis, or underlying cause of death?	◯ Communicable and nutritional conditions<br>◯ Non-communicable diseases<br>◯ Injuries<br>◯ Ill-defined or cause unknown
Choose the most appropriate diagnosis	◯ Pneumonia<br>◯ Bronchiolitis<br>◯ Upper respiratory tract infection or croup<br>◯ Tuberculosis<br>◯ Diarrhea/gastroenteritis<br>◯ Hepatitis<br>◯ Measles<br>◯ Pertussis<br>◯ Tetanus<br>◯ Urinary tract infection or pyelonephritis<br>◯ Acute otitis media<br>◯ Pharyngitis<br>◯ HIV/AIDS or AIDS-related illness<br>◯ Sepsis or septic shock<br>◯ Acute Malaria<br>◯ Multisystem Inflammatory Syndrome in Children (MISC)<br>◯ Acute COVID-19<br>◯ Any skin or soft tissue infection<br>◯ Malnutrition<br>◯ Meningitis or Encephalitis<br>◯ Fever and neutropenia<br>◯ Other infectious or parasitic disease
Please indicate specific forms of malnutrition (select all that apply)	☐ Wasting<br>☐ Failure to thrive<br>☐ Kwashiorkor<br>☐ Severe acute malnutrition (SAM)<br>☐ Stunting<br>☐ Marasmus<br>☐ Other<br>☐ Not documented
List other type of malnutrition	______________________________
If OTHER infectious or parasitic disease, please describe	_____________________________________

Choose the most appropriate diagnosis	◯ Congenital malformations<br>◯ Birth Asphyxia<br>◯ Prematurity<br>◯ Hydrocephalus (with or without a VPS)<br>◯ Stroke<br>◯ Status Epilepticus or seizure<br>◯ Heart Failure<br>◯ Diabetes or related complication (diabetic ketoacidosis, hyperglycemia, hypoglycemia)<br>◯ Bowel obstruction<br>◯ Intussusception<br>◯ Appendicitis<br>◯ Gastrointestinal bleed (upper or lower)<br>◯ Peptic ulcer disease/GERD/Reflux<br>◯ Constipation<br>◯ Pancreatitis<br>◯ Cancer/malignancy<br>◯ Allergies, allergic rhinitis<br>◯ Asthma/Status Asthmaticus<br>◯ Chronic Respiratory or lung Disease<br>◯ Sickle cell disease/anemia or associated complication (acute chest, pain crisis)<br>◯ Hypovolemia/Dehydration<br>◯ Shock<br>◯ Anemia<br>◯ Renal failure or injury<br>◯ Carbon monoxide poisoning<br>◯ Other non-communicable diseases
What is the cancer or oncologic diagnosis?	__________________________________
Indicate whether acute or chronic kidney or renal injury	◯ Acute<br>◯ Chronic<br>◯ Not documented
Indicate the type of shock	◯ Cardiogenic<br>◯ Neurogenic<br>◯ Anaphylactic<br>◯ Hemorrhagic<br>◯ Hypovolemic due to dehydration<br>◯ Obstructive<br>◯ Other<br>◯ Not documented
Other type of shock	__________________________________
Indicate the type of stroke	◯ Non-traumatic hemorrhagic stroke<br>◯ Ischemic<br>◯ Other<br>◯ Not documented
List or describe other kind of stroke	__________________________________

If OTHER non-communicable disease, please describe

____

Choose the most appropriate diagnosis

- ◯ Traumatic brain injury
- ◯ Polytrauma
- ◯ Fracture
- ◯ Laceration
- ◯ Non-accidental trauma or child abuse
- ◯ Self-injury or suicide attempt
- ◯ Assault
- ◯ Fall
- ◯ Drowning
- ◯ Poisoning/Ingestion
- ◯ Burn
- ◯ Envonmation by either bite or sting
- ◯ Foreign body aspiration
- ◯ Foreign body ingestion
- ◯ Other injury

If OTHER injury, please describe

____

If ill-defined, please describe

____

Would you like to add a SECONDARY diagnosis

- ◯ Yes
- ◯ No

Choose the most appropriate SECONDARY diagnosis

- ◯ Communicable and nutritional conditions
- ◯ Non-communicable diseases
- ◯ Injuries
- ◯ Ill-defined or cause unknown

Choose the most appropriate diagnosis

- ◯ Pneumonia
- ◯ Bronchiolitis
- ◯ Upper respiratory tract infection or croup
- ◯ Tuberculosis
- ◯ Diarrhea/gastroenteritis
- ◯ Hepatitis
- ◯ Measles
- ◯ Pertussis
- ◯ Tetanus
- ◯ Urinary tract infection or pyelonephritis
- ◯ Acute otitis media
- ◯ Pharyngitis
- ◯ HIV/AIDS or AIDS-related illness
- ◯ Sepsis or septic shock
- ◯ Acute Malaria
- ◯ MISC
- ◯ Acute COVID-19
- ◯ Any skin or soft tissue infection
- ◯ Malnutrition
- ◯ Meningitis or Encephalitis
- ◯ Fever and neutropenia
- ◯ Other infectious or parasitic disease

Type of malnutrition, choose all that apply	☐ Wasting<br>☐ Failure to thrive<br>☐ Kwashiorkor<br>☐ Severe acute malnutrition (SAM)<br>☐ Stunting<br>☐ Marasmus<br>☐ Other<br>☐ Not documented
List other type of malnutrition	__________________________________
Please give details for other infectious cause or parasitic disease	__________________________________
Choose the most appropriate diagnosis	◯ Congenital malformations<br>◯ Birth Asphyxia<br>◯ Prematurity<br>◯ Hydrocephalus (with or without a VPS)<br>◯ Stroke<br>◯ Status Epilepticus or seizure<br>◯ Heart Failure<br>◯ Diabetes or related complication (diabetic ketoacidosis, hyperglycemia, hypoglycemia)<br>◯ Bowel obstruction<br>◯ Intussusception<br>◯ Appendicitis<br>◯ Gastrointestinal bleed (upper or lower)<br>◯ Peptic ulcer disease/GERD/Reflux<br>◯ Constipation<br>◯ Pancreatitis<br>◯ Cancer/malignancy<br>◯ Allergies, allergic rhinitis<br>◯ Asthma/Status Asthmaticus<br>◯ Chronic Respiratory or lung Disease<br>◯ Sickle cell disease/anemia or associated complication (acute chest, pain crisis)<br>◯ Hypovolemia/Dehydration<br>◯ Shock<br>◯ Anemia<br>◯ Renal failure or injury<br>◯ Carbon monoxide poisoning<br>◯ Other non-communicable diseases
Indicate type of stroke	◯ Non-traumatic hemorrhagic stroke<br>◯ Ischemic<br>◯ Other<br>◯ Not documented
Indicate other stroke type	__________________________________
Indicate cancer or malignancy diagonsis	__________________________________

Indicate shock type	◯ Cardiogenic<br>◯ Neurogenic<br>◯ Anaphylactic<br>◯ Hemorrhagic<br>◯ Hypovolemic due to dehydration<br>◯ Obstructive<br>◯ Other<br>◯ Not documented
Indicate other shock type	__________________________________
Indicate whether acute or chronic kidney or renal injury	◯ Acute<br>◯ Chronic<br>◯ Not documented
If OTHER, please describe	__________________________________
Choose the most appropriate diagnosis	◯ Traumatic brain injury<br>◯ Polytrauma<br>◯ Fracture<br>◯ Laceration<br>◯ Non-accidental trauma or child abuse<br>◯ Self-injury or suicide attempt<br>◯ Assault<br>◯ Fall<br>◯ Drowning<br>◯ Poisoning/Ingestion<br>◯ Burn<br>◯ Envonmation by either bite or sting<br>◯ Foreign body aspiration<br>◯ Foreign body ingestion<br>◯ Other injury
If OTHER, please describe	__________________________________
If ill-defined, describe	__________________________________
Would you like to add a TERTIARY diagonsis	◯ Yes<br>◯ No
TERTIARY Diagnosis	◯ Communicable and nutritional conditions<br>◯ Non-communicable diseases<br>◯ Injuries<br>◯ Ill-defined or cause unknown

Choose the most appropriate	◯ Pneumonia<br>◯ Bronchiolitis<br>◯ Upper respiratory tract infection or croup<br>◯ Tuberculosis<br>◯ Diarrhea/gastroenteritis<br>◯ Hepatitis<br>◯ Measles<br>◯ Pertussis<br>◯ Tetanus<br>◯ Urinary tract infection or pyelonephritis<br>◯ Acute otitis media<br>◯ Pharyngitis<br>◯ HIV/AIDS or AIDS-related illness<br>◯ Sepsis or septic shock<br>◯ Acute Malaria<br>◯ MISC<br>◯ Acute COVID-19<br>◯ Any skin or soft tissue infection<br>◯ Malnutrition<br>◯ Meningitis or Encephalitis<br>◯ Fever and neutropenia<br>◯ Other
Indicate type of malnutrition, choose all that apply.	☐ Wasting<br>☐ Failure to thrive<br>☐ Kwashiorkor<br>☐ Severe acute malnutrition (SAM)<br>☐ Stunting<br>☐ Marasmus<br>☐ Other<br>☐ Not documented
Other type of malnutrition	__________________________________
If other, please describe	__________________________________

Choose the most appropriate diagnosis	◯ Congenital malformations<br>◯ Birth Asphyxia<br>◯ Prematurity<br>◯ Hydrocephalus (with or without a VPS)<br>◯ Stroke<br>◯ Status Epilepticus or seizure<br>◯ Heart Failure<br>◯ Diabetes or related complication (diabetic ketoacidosis, hyperglycemia, hypoglycemia)<br>◯ Bowel obstruction<br>◯ Intussusception<br>◯ Appendicitis<br>◯ Gastrointestinal bleed (upper or lower)<br>◯ Peptic ulcer disease/GERD/Reflux<br>◯ Constipation<br>◯ Pancreatitis<br>◯ Cancer/malignancy<br>◯ Allergies, allergic rhinitis<br>◯ Asthma/Status Asthmaticus<br>◯ Chronic Respiratory or lung Disease<br>◯ Sickle cell disease/anemia or associated complication (acute chest, pain crisis)<br>◯ Hypovolemia/Dehydration<br>◯ Shock<br>◯ Anemia<br>◯ Renal failure or injury<br>◯ Carbon monoxide poisoning<br>◯ Other non-communicable diseases
Indicate the type of stroke	◯ Non-traumatic hemorrhagic stroke<br>◯ Ischemic<br>◯ Other<br>◯ Not documented
Indicate other stroke type	__________________________________
Indicate cancer or oncologic diagnosis	__________________________________
Indicate the type of shock	◯ Cardiogenic<br>◯ Neurogenic<br>◯ Anaphylactic<br>◯ Hemorrhagic<br>◯ Hypovolemic due to dehydration<br>◯ Obstructive<br>◯ Other<br>◯ Not documented
Indicate other shock type	__________________________________
Indicate whether acute or chronic kidney or renal injury	◯ Acute<br>◯ Chronic<br>◯ Not documented

If OTHER, please describe

____

Choose the most appropriate diagnosis

◯ Traumatic brain injury
◯ Polytrauma
◯ Fracture
◯ Laceration
◯ Non-accidental trauma or child abuse
◯ Self-injury or suicide attempt
◯ Assault
◯ Fall
◯ Drowning
◯ Poisoning/Ingestion
◯ Burn
◯ Envonmation by either bite or sting
◯ Foreign body aspiration
◯ Foreign body ingestion
◯ Other injury

If OTHER, please describe

____

If ill-defined, please describe

____

If acute COVID-19 infection of MIS-C associated with COVID-19, how was it diagonsed?

◯ Viral PCR or NAAT (nose swab)
◯ Other rapid test (antigen)
◯ COVID antibody positive
◯ Close association with others with COVID-19

Co-Morbid Conditions

For all comorbid conditions, select the most appropriate conditions that best match conditions listed in the problem list or past medical history

Asthma/reactive airways disease

◯ Yes
◯ No/No documented history

Congential Heart Disease

◯ Yes
◯ No/No documented history
◯ Suspected but unable to confirm

Select specific congenital heart lesion, if known. Choose all that apply.

☐ Ventricular septal defect (VSD)
☐ Atrial septal defect (ASD)
☐ Tetralogy of Fallot (TOF)
☐ Patent ductus arteriosus (PDA)
☐ Truncus arteriosus (TA)
☐ Transposition of the great arteries (TGA)
☐ Total or partial anomalous venous drainage
☐ Undifferentiated "cyanotic" heart disease
☐ Undifferentiated "acyanotic" heart disease
☐ Other

If "other" type of congenital heart disease, note it here

____

Rheumatic Heart Disease	◯ Yes<br>◯ No/No documented history
Human Immunodeficiency Virus (HIV)	◯ Negative<br>◯ Positive<br>◯ Exposed<br>◯ Unknown/Not documented
Malnutrition	◯ Yes<br>◯ No/No documented history
Indicate malnutrition type	☐ Wasting<br>☐ Failure to thrive<br>☐ Kwashiorkor<br>☐ Severe acute malnutrition (SAM)<br>☐ Stunting<br>☐ Marasmus<br>☐ Other<br>☐ Not documented
Indicate other type of malnutrition	________________________________
Cancer/malignancy	◯ Yes<br>◯ No/No documented history
What is the cancer diagnosis	__________________________
Obesity	◯ Yes<br>◯ No/No documented history
Diabetes	◯ Yes<br>◯ No/No documented history
Developmental Delay	◯ Yes<br>◯ No/No documented history
Cerebral Palsy	◯ Yes<br>◯ No/No documented history
Seizure disorder or epilepsy or fits	◯ Yes<br>◯ No/No documented history
Hydrocephalus	◯ Yes<br>◯ No/No documented history
Sickle cell disease/anemia	◯ Yes<br>◯ No/No documented history
Thalassemia	◯ Yes<br>◯ No/No documented history
Hypertension	◯ Yes<br>◯ No/No documented history

Genetic or congenital condition	◯ Yes<br>◯ No/No documented history
Please describe any genetic or congenital conditions	__________________________________
Any other co-morbid condition not listed above?	◯ Yes<br>◯ No
Any other co-morbid condition?<br><br>Add one per field	__________________________________
Would you like to add another comorbid condition?	◯ Yes<br>◯ No
Any other co-morbid condition?<br><br>Add one per field	__________________________________
Would you like to add another comorbid condition?	◯ Yes<br>◯ No
Any other co-morbid condition?<br><br>Add one per field	__________________________________
Would you like to add another comorbid condition?	◯ Yes<br>◯ No
Any other co-morbid condition?<br><br>Add one per field	__________________________________
Any additional comments	__________________________________
